# Efficacy of immunonutritional supplement after neoadjuvant chemotherapy in patients with esophageal cancer

**DOI:** 10.1186/s13019-022-01786-x

**Published:** 2022-03-19

**Authors:** Chao Luo, Kai Xie, Chi Zhang, Zhuang-Zhuang Cong, Wen-Feng Gu, Yang Xu, Yong Qiang, Xiao-Kun Li, Chao Zheng, Li-Wen Hu, Yi Shen

**Affiliations:** 1 Department of Cardiothoracic Surgery, Jinling Hospital, Southern Medical University, Guangzhou, China; 2 Department of Cardiothoracic Surgery, Jinling Hospital, School of Clinical Medicine, Nanjing Medical University, Nanjing, China; 3 Department of Cardiothoracic Surgery, Jinling Hospital, Medical School of Nanjing University, Nanjing, China; 4 Department of Cardiothoracic Surgery, Jinling Hospital, School of Medicine, Southeast University, Nanjing, China

**Keywords:** Esophageal cancer, Neoadjuvant chemotherapy, Immunonutrition, Nutritional status

## Abstract

**Background:**

In recent years, preoperative nutrition has received great attention, especially for patients who received surgical reconstruction of the digestive tract such as esophagectomy. Preoperative nutrition therapy was reported to accelerate the patient's postoperative recovery. In addition, immune suppression, nausea, and vomiting may lead to poor immune and nutritional status of patients with esophageal cancer who underwent neoadjuvant chemotherapy (NAC), which is not conducive to surgery. Therefore, preoperative nutritional treatment is necessary for patients with esophageal cancer who underwent NAC.

**Method:**

Patients with esophageal cancer who received NAC at Nanjing Jinling Hospital from January 2018 to September 2020 were retrospectively identified. Patients were divided into enteral immunonutrition (EIN) group (those who received a conventional diet and immunonutrition supplement, Peptisorb, Nutricia, 500 mL/day * 7 via oral intake), and control group (those who only received a conventional diet were divided into). The primary outcomes were immune and nutritional indicators changes, including immunoglobulin M (IgM), immunoglobulin A (IgA), immunoglobulin G (IgG), and albumin (ALB), which were measured at preoperative day (PRD) 7, PRD-1, postoperative day (POD) 1 and POD-7. The secondary outcomes were postoperative complications, adverse reactions, and length of hospital stay.

**Results:**

A total of 124 eligible patients were included in the study, with 21 patients in EIN group. After 1:2 matching, significant difference in baseline characteristics between the two groups was not observed (EIN: n = 21, Control group: n = 42). Compared with the control group, the IgA is significantly increased in the EIN group at POD-7 (*p* = 0.017). However, we observed that the IgM level in the control group was significantly higher than those in the EIN group at POD-7 (*p* = 0.007). The incidence of pneumonia and total complications in the EIN group were significantly lower than those in control group (*p* = 0.024, *p* = 0.028, respectively). There is no significant difference in ALB and adverse reactions between two groups (*p* = 0.303, *p* = 0.108, respectively).

**Conclusion:**

Immunonutritional supplement after NAC is an effective strategy to improve the postoperative immune status of esophageal cancer patients and could reduce the incidence of infectious complication. More well-designed prospective studies are needed to verify and update our finding.

**Supplementary Information:**

The online version contains supplementary material available at 10.1186/s13019-022-01786-x.

## Introduction

Esophageal cancer is one of the most common tumors and ranks sixth in mortality worldwide [1]. NAC combined with surgery is one of the common treatments for patients with locally advanced esophageal cancer [2]. For these patients, NAC may significantly enable them to obtain a better overall survival rate [3]. However, NAC may lead to a delay in surgery and an amplification of operative and postoperative complications [3, 4]. Dysphagia and weight loss in patients with esophageal cancer will lead to malnutrition. Nausea and vomiting often occur during the period of NAC, which will aggravate malnutrition of patients and is not conducive to the follow-up treatment of patients. It is reported that preoperative malnutrition will also affect the postoperative prognosis, including complications and adverse reactions [5, 6]. The interval between NAC and surgery provides the possibility to correct the deterioration of nutritional status before esophagectomy [7]. Immune-modulating substances such as arginine, omega-3 fatty acids, and antioxidants can modulate immune and inflammatory processes in major surgery and improve clinical outcomes. Arginine is a non-essential amino acid that plays a role in the synthesis of nitric oxide, which regulates gene expression and stimulates cell-mediated immunity [8].

However, there are few studies to explore the effect of immunonutrition therapy/supplement after NAC in patients with esophageal cancer. So, we conducted a retrospective study to explore the effects of preoperative immunonutritional support after NAC on postoperative immunonutritional parameters, postoperative complication, adverse reaction, and length of hospital stay in patients with esophageal cancer.

## Materials and methods

### Patients

**Fig. 1:**
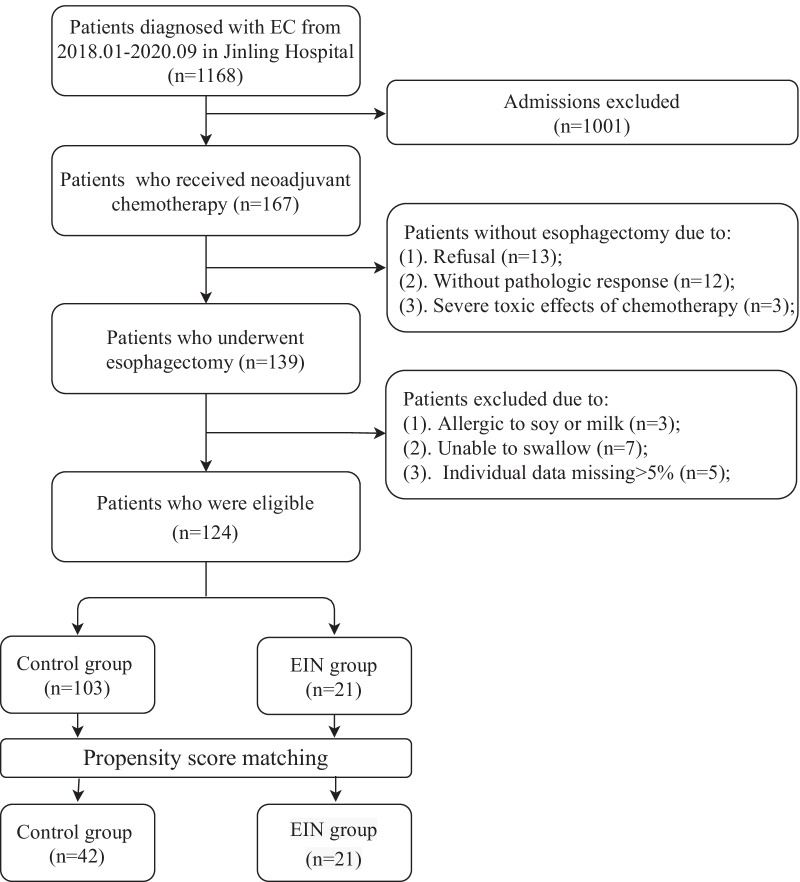
Flow chart of participants selection

Between 2018.01 and 2020.09, 1168 was diagnosed with esophageal cancer in Jinling hospital (Fig. 1). 1001 patients who didn’t receive neoadjuvant chemotherapy were excluded. Twenty-eight patients were withdrawn without esophagectomy. Of the remained 139 patients underwent esophagectomy, 15 patients were excluded due to allergic to soy or milk, unable to swallow, or individual data missing > 5%. Finally, 124 patients were eligible for the study. 103 patients in the control group and 21 patients in the EIN group were analyzed. A propensity score matching analysis was performed to reduce the effects of characteristics. Finally, 42 patients were included in the control group and 21 patients were included in the EIN group.

### Study design

A retrospective study was performed between January 1, 2018, and September 1, 2020, at the Department of Cardiothoracic Surgery, Jinling Hospital, Nanjing, China. All patients included in the study received two courses of NAC. The chemotherapy regimen was paclitaxel plus cisplatin. Patients in the EIN group received enteral immune nutrition fluids (500 mL/day, Enteral Nutritional Emulsion, ω-3 Fatty Acids, Arginine) with their regular meals consecutively for a week after NAC, and the control group received only regular meals consecutively for a week after NAC. The primary endpoints were immune and nutritional parameters, including IgM, IgA, IgG, and ALB, which were measured at PRD-7, PRD-1, POD-1, and POD-7. The secondary endpoints were, complications, adverse reactions, and length of hospital stay. Postoperative complications included anastomotic fistula, pneumonia, chylothorax, and recurrent laryngeal nerve injury. All postoperative complications were evaluated according to Clavien-Dindo criteria and grade II or more was defined as positive [9]

Meanwhile, baseline characteristics of all included patients were extracted from the hospital information system (HIS), including age, sex, body mass index (BMI), comorbidities, TNM stage, tumor site, differentiation grade, size. Tumor staging was based on the seventh edition of the International Union for Cancer Control (UICC) TNM staging system for esophageal carcinoma.

### Objects of study

The inclusion criteria were as follows: (1) Patients who received NAC (2) Patients who underwent esophagectomy. Patients were excluded when: (1) Patients without esophagectomy due to: (1) Refusal; (2) Without pathologic response; (3) Severe toxic effects of chemotherapy; (2) Patients excluded due to: (1) Allergic to soy or milk; (2) Unable to swallow; (3) Individual data missing > 5%.

### Statistical analyses

Continuous variables were performed as the mean with standard deviation (SD) or median with interquartile ranges (IQR). The student t-test, χ^2^ tests or Fisher’s exact tests were utilized to compare the difference between the two groups. We created a propensity score matched cohort by attempting to match each patient receiving enteral immune nutrition fluids with their regular meals with a patient receiving regular meals (1:2 match). The covariates included in the propensity score matching were age, sex, BMI, diabetes, hypertension, smoking, and alcohol. All statistical analyses were executed with Stata 16.0 (Stata Corp LLC, college station, USA). *P* < 0.05 was considered statistically significant.

## Results

As showed in Fig. 1, a total of 1168 patients were screened and 1001 patients who didn’t receive neoadjuvant chemotherapy were excluded. Twenty-eight patients were withdrawn without esophagectomy. Of the remained 139 patients underwent esophagectomy, 15 patients were excluded due to allergic to soy or milk, unable to swallow, or individual data missing > 5%. Finally, 124 patients were eligible for the study. 103 patients in the control group and 21 patients in the EIN group were analyzed. After a propensity score matching, 42 patients were included in the control group and 21 patients were included in the EIN group.

The baseline characteristics of patients were presented in Table 1. Significant differences were not observed between the two groups after propensity score matching.

**Table 1:** Comparison of baseline characteristics between EIN group and control group

Characteristics	Before matching			After matching		
	Control group (n = 103)	EIN group (n = 21)	*p* value	Control group (n = 42)	EIN group (n = 21)	*p* value
Age(years)	63.58 ± 7.2	61.95 ± 7.68	0.352	61.02 ± 7.25	61.95 ± 7.68	0.649
Sex			0.885			0.496
Man	87	18		33	18	
Female	16	3		9	3	
BMI (kg/m^2^)	23.48 ± 3.56	21.92 ± 3.50	0.068	21.7 ± 3.201	21.92 ± 3.50	0.855
Diabetes			0.661			0.595
Yes	7	2		2	2	
No	96	19		40	19	
Hypertension			0.179			> 0.999
Yes	35	4		9	4	
No	68	17		33	17	
Smoking			0.417			> 0.999
Yes	59	10		20	10	
No	44	11		22	11	
Alcohol			0.795			0.858
Yes	62	12		23	12	
No	41	9		19	9	
Type of operation			0.327			0.858
Minimal invasive	61	10		19	10	
Open	42	11		23	11	
Operation time (h)	3.73 ± 0.83	4.03 ± 0.82	0.133	3.63 ± 0.749	4.03 ± 0.82	0.057
T stage			0.650			0.759
T1	8	1		3	1	
T2	17	5		6	5	
T3	57	9		24	9	
T4	5	2		3	2	
Unknown	16	4		6	4	
N stage			0.917			0.538
N0	42	7		21	7	
N1	30	7		11	7	
N2	17	4		7	4	
N3	3	0		0	0	
4 (unknown)	11	3		3	3	
M stage	0	0			0	
Tumor site			0.555			0.206
Upper	8	1		3	1	
Middle	63	11		30	11	
Lower	32	9		9	9	
Degree of differentiation			0.338			0.627
Well	13	5		8	5	
Moderately	66	13		23	13	
Poorly	24	3		11	3	
Size (cm)	3.67 ± 1.63	3.83 ± 1.76	0.671	3.18 ± 1.48	3.83 ± 1.76	0.126

BMI, body mass index; EIN, enteral immunonutrition; Minimal invasive: video-assisted thoracic surgery and Da Vinci robot assisted surgery

Immune and nutritional indicator of all patients were measured at the four timepoints mentioned above shown in Table 2. Immune indicators (IgA) in the two groups have significantly differences at POD-7 (*p* = 0.017). However, patients in the control group have a higher level of IgM at POD-7 (*p* = 0.007). Nutritional indicator (ALB) in two groups has no significant difference at POD-7.

**Table 2:** Comparison of immune and nutritional parameters

Items	Before matching			After matching		
	Control group (n = 103)	EIN group (n = 21)	*p* value	Control group (n = 42)	EIN group (n = 21)	*p* value
IgM						
PRD-7	1.27 ± 0.42	1.26 ± 0.42	0.926	1.30 ± 0.40	1.26 ± 0.42	0.662
PRD-1	1.29 ± 0.50	1.28 ± 0.53	0.962	1.27 ± 0.50	1.28 ± 0.53	0.896
POD-1	1.03 ± 0.32	0.99 ± 0.50	0.643	1.05 ± 0.37	0.99 ± 0.50	0.619
POD-7	1.5 ± 0.35	1.18 ± 0.33	0.001	1.43 ± 0.33	1.18 ± 0.33	0.007
IgA						
PRD-7	2.21 ± 0.55	2.20 ± 0.58	0.904	2.21 ± 0.55	2.20 ± 0.58	0.880
PRD-1	2.23 ± 0.67	2.65 ± 0.72	0.109	2.48 ± 0.620	2.65 ± 0.72	0.356
POD-1	1.94 ± 0.59	1.93 ± 0.62	0.934	1.92 ± 0.70	1.93 ± 0.62	0.991
POD-7	2.13 ± 0.65	2.44 ± 0.65	0.045	2.02 ± 0.65	2.44 ± 0.65	0.017
IgG						
PRD-7	11.65 ± 2.5	11.28 ± 2.53	0.538	11.39 ± 2.51	11.28 ± 2.53	0.866
PRD-1	12.23 ± 2.08	12.72 ± 1.93	0.320	12.31 ± 1.98	12.72 ± 1.93	0.432
POD-1	10.48 ± 1.89	10.78 ± 1.7	0.497	10.53 ± 1.77	10.78 ± 1.7	0.591
POD-7	11.02 ± 1.81	11.90 ± 1.77	0.042	11.43 ± 1.76	11.90 ± 1.77	0.315
ALB						
PRD-7	34.75 ± 3.99	33.95 ± 4.12	0.407	35.67 ± 4.52	33.95 ± 4.12	0.148
PRD-1	40.19 ± 3.96	39.99 ± 4.64	0.837	41.16 ± 4.01	39.99 ± 4.64	0.303
POD-1	33.42 ± 3.46	33.55 ± 3.33	0.876	33.54 ± 4.09	33.55 ± 3.33	0.991
POD-7	35.14 ± 4.18	35.11 ± 3.60	0.981	33.926 ± 4.58	35.11 ± 3.60	0.303

EIN, enteral immunonutrition, IgM, immunoglobulin M. IgA, immunoglobulin A, IgG, immunoglobulin G, ALB, albumin. PRD, preoperative days. POD, postoperative days

The secondary outcomes are listed in Table 3. The incidence of pneumonia and total complications in the EIN group were significantly lower than those in control group (*p* = 0.024, *p* = 0.028, respectively). There were no significant differences in the hospital stays and the incidence of adverse reaction between two groups.

**Table 3:** Comparison of surgical outcomes between two groups

	Before matching			After matching		
Variables	Control group (n = 103)	EIN group (n = 21)	*p* value	Control group (n = 42)	EIN group (n = 21)	*p* value
Complication						
All	42	4	0.600	20	4	0.028
Anastomotic fistula	11	2	0.999	3	2	> 0.999
Pneumonia	26	1	0.043	13	1	0.024
Others	5	1	0.999	4	1	0.657
Hospital stays (day)	15 (13–17)	13 (11–16)	0.039	15 (13–16)	13 (11–16)	0.081
Adverse reactions						
Bloating and diarrhea	26	3	0.280	14	3	0.108

EIN, enteral immunonutrition, Others, chylothorax and recurrent laryngeal nerve injury

Linear regression of anastomotic leakage (AL) and nutritional indicators are showed in the Additional file 1: Table S1. Anastomotic leakage has statistical significance for ALB at POD-1 (*p* = 0.038), which should be discarded. Because anastomotic leakage generally does not occur on the first day after operation. There was no statistical significance in the nutritional way for ALB at POD-1 and POD-7. And anastomotic leakage has no statistical significance for IgM, IgA, IgG at POD-7.

Logistic regression of type of operation and complications are showed in the Additional file 1: Table S2. Type of operation has no statistical significance for anastomotic leakage and pneumonia.

## Discussion

Esophageal cancer is a common gastrointestinal tumor in the world. There are many treatment options for advanced esophageal cancer, one of which is neoadjuvant chemotherapy (NAC) combined with esophagectomy. Multimodality treatment, which implies NAC in combination with surgery, has shown a better survival benefit than surgery alone [10–12]. Malnutrition is highly prevalent in patients with esophageal cancer. Due to the influence of tumor, patients with esophageal cancer often have symptoms such as dysphagia, vomiting, insufficient nutritional intake, fatigue, weight, and muscle loss. The adverse effects of NAC, such as immune suppression, nausea and vomiting, combined with the clinical symptoms of esophageal tumor itself, will adversely affect the follow-up treatment. It’s very important to integrate the early screening of malnutrition and appropriate nutritional support into the overall preoperative management of esophageal cancer patients [13–16]. According to previous studies, preoperative nutritional support can improve postoperative nutritional status and immune function [17, 18].

In our study, immunoglobulin for patients with EIN was found to outstrip those who are without EIN. IgA was significantly higher in the EIN group (*p* = 0.017) at POD-7. IgM in the control group is significantly higher than the EIN group (*p* = 0.007). Although there is fewer differences in IgG between two groups from POD-1 to POD-7, IgG is still better in the EIN group.

Immunoglobulin plays an important role in the humoral immune system. IgA is divided into serum type and secretory type; the secretory type is the main antibody in mucosal infection. Patients with esophageal cancer always suffer from eating disorders for a long time which might cause intestinal mucosal barrier dysfunction [19]. EIN may be beneficial to maintain the integrity of the structure and function of intestinal mucosal cells, and to protect the intestinal mucosal barrier [20]. IgM appears first in infection. In control group, the incidence of pneumonia is higher than the EIN group. The high incidence of pneumonia in the control group could result in the high level of IgM, compared with the EIN group. Thus, EIN may be beneficial for patients with esophageal cancer after NAC by enhancing their immune function and reducing the incidence of infectious complication and length of hospital stay. However, some researchers uphold that there are no significant differences in clinical outcomes (complications, hospital stay) between treatment and control group, that used immunonutrition before esophageal cancer surgery [21–25]. But Alfred Adiamah et al. [26] thought that preoperative immunonutrition could lead to an appreciable and significant reduction in postoperative infectious complications and a tendency for a shortened length of hospital stay. The conclusion is consistent with our result. But the systematic review and meta-analysis included most gastrointestinal cancers but few esophageal cancers.

### Limitation

It is a retrospective single-center study that may be subject to selection bias. And this is a small-scale study which may lead to unstable results; therefore, we will continue to conduct further studies with larger samples in this area to prove significant differences between the two groups. We did not conduct Kaplan–Meier curves to compare the difference in long-term survival between groups.

## Conclusion

Immunonutritional supplement after NAC is an effective strategy to improve the postoperative immune status of esophageal cancer patients and reduce the incidence of infectious complication and length of hospital stay. More well-designed prospective studies are needed to verify and update our finding.

## Supplementary Information

**Additional file 1: Table S1.** linear regression of anastomotic leakage (AL) and nutritional indicators. **Table S2.** logistic regression of type of operation and complications.
