# Supplementary material for: Efficacy of immunonutritional supplement after neoadjuvant chemotherapy in patients with esophageal cancer
Source: J Cardiothorac Surg. 2022 Mar 19;17:41. doi: 10.1186/s13019-022-01786-x (PMC8933903; doi:10.1186/s13019-022-01786-x)
Supplement: Supplementary file 1 — Additional file 1: Table S1. linear regression of anastomotic leakage (AL) and nutritional indicators. Table S2. logistic regression of type of operation and complications. [file 13019_2022_1786_MOESM1_ESM.docx]

**Supplement table 1.** linear regression of anastomotic leakage (AL) and nutritional indicators.

| Items |  | β | 95%CI | p value |
| --- | --- | --- | --- | --- |
| ALB |  |  |  |  |
| POD-1 | Nutritional way | 0.1 | -1.90-2.11 | 0.921 |
|  | AL | -3.71 | -7.21--0.20 | 0.038 |
| POD-7 | Nutritional way | 1.24 | -1.04-3.53 | 0.280 |
|  | AL | -2.35 | -6.33-1.63 | 0.242 |
| IgM |  |  |  |  |
| POD-1 | Nutritional way | -0.06 | -0.28-0.16 | 0.592 |
|  | AL | 0.19 | -0.21-0.58 | 0.346 |
| POD-7 | Nutritional way | 0.25 | 0.67-0.43 | 0.008 |
|  | AL | -0.02 | -0.34-0.30 | 0.889 |
| IgA |  |  |  |  |
| POD-1 | Nutritional way | 0.01 | -0.34-0.37 | 0.945 |
|  | AL | -0.44 | -1.06-0.18 | 0.165 |
| POD-7 | Nutritional way | 0.42 | 0.07-0.78 | 0.019 |
|  | AL | 0.08 | -0.53-0.70 | 0.786 |
| IgG |  |  |  |  |
| POD-1 | Nutritional way | 0.22 | -0.70-1.13 | 0.638 |
|  | AL | 1.49 | -0.11-3.09 | 0.067 |
| POD-7 | Nutritional way | 0.50 | -0.44-1.44 | 0.289 |
|  | AL | -1.04 | -2.68-0.60 | 0.209 |

**Supplement table 2.** logistic regression of type of operation and complications

| Complications |  | OR | 95%CI | p value |
| --- | --- | --- | --- | --- |
| AL | Nutritional way | 1.34 | 0.20-9.05 | 0.766 |
|  | Type of operation | 5.26 | 0.55-50.04 | 0.149 |
| pneumonia | Nutritional way | 0.11 | 0.01-0.91 | 0.041 |
|  | Type of operation | 1.30 | 0.37-4.52 | 0.682 |
